# The effectiveness of critical time intervention for abused women and homeless people leaving Dutch shelters: study protocol of two randomised controlled trials

**DOI:** 10.1186/1471-2458-13-555

**Published:** 2013-06-06

**Authors:** Danielle AM Lako, Renée de Vet, Mariëlle D Beijersbergen, Daniel B Herman, Albert M van Hemert, Judith RLM Wolf

**Affiliations:** 1 Department of Primary and Community Care, Radboud University Nijmegen Medical Centre, Geert Grooteplein 21, Nijmegen, The Netherlands; 2 Silberman School of Social Work, Hunter College, 2180 Third Avenue, New York, NY, USA; 3 Department of Psychiatry, Leiden University Medical Centre, Albinusdreef 2, Leiden, The Netherlands

**Keywords:** Critical time intervention, RCT, Intimate partner violence, Homelessness, Shelters

## Abstract

**Background:**

One of the main priorities of Dutch organisations providing shelter services is to develop evidence-based interventions in the care for abused women and homeless people. To date, most of these organisations have not used specific intervention models and the interventions which have been implemented rarely have an empirical and theoretical foundation. The present studies aim to examine the effectiveness of critical time intervention (CTI) for abused women and homeless people.

**Methods:**

In two multi-centre randomised controlled trials we investigate whether CTI, a time-limited (nine month) outreach intervention, is more effective than care-as-usual for abused women and homeless people making the transition from shelter facilities to supported or independent housing. Participants were recruited in 19 women’s shelter facilities and 22 homeless shelter facilities across The Netherlands and randomly allocated to the intervention group (CTI) or the control group (care-as-usual). They were interviewed four times in nine months: once before leaving the shelter, and then at three, six and nine months after leaving the shelter. Quality of life (primary outcome for abused women) and recurrent loss of housing (primary outcome for homeless people) as well as secondary outcomes (e.g. care needs, self-esteem, loneliness, social support, substance use, psychological distress and service use) were assessed during the interviews. In addition, the model integrity of CTI was investigated during the data collection period.

**Discussion:**

Based on international research CTI is expected to be an appropriate intervention for clients making the transition from institutional to community living. If CTI proves to be effective for abused women and homeless people, shelter services could include this case management model in their professional standards and improve the (quality of) services for clients.

**Trial registration:**

NTR3463 and NTR3425

## Background

Although abused women and homeless people leaving shelters represent different subgroups of vulnerable people in society, they share a crucial similarity: during the critical period of transition from shelter to community living they are both at a substantially increased risk for recurrence of the adverse events which brought them to the shelters in the first place [1,2]. Institutional discharge has proved to be challenging for socially vulnerable people: they have to deal with a complex and fragmented system of care and are at a high risk of experiencing a loss of personal contacts during this transition period [3,4].

Shelters for abused women and homeless people aim to prevent relapse of clients into their former situation and provide services to improve clients’ well-being and support their integration into the community [5]. However, shelter organisations do not use standardised interventions which have been proved to be effective in supporting clients when they leave [6,7]. The need for such interventions has increased in light of recent requirements by local authorities and health insurance companies to be accountable for care trajectories of clients and outcomes [8].

To date, studies into the effectiveness of interventions have been conducted mostly in the United States, and little is known about whether these evidence-based interventions would also be effective in European shelter systems (De Vet, Van Luijtelaar, Brilleslijper-Kater, Vanderplasschen, Beijersbergen & Wolf, unpublished data, May, 2013; Jonker, Sijbrandij, Van Luijtelaar, Cuijpers & Wolf, unpublished data, December, 2012) [6,9]. The present studies examine whether a time-limited outreach intervention, critical time intervention (CTI) [10], is more effective than care-as-usual for abused women and homeless people who are making the transition from a shelter facility to supported or independent housing in The Netherlands.

CTI is being applied and tested in the United States, the United Kingdom and Brazil [4] and has been investigated among a range of populations, such as men and women after discharge from inpatient psychiatric treatment [11,12] and people being released from prison [13,14]. Research among homeless men with serious mental illness has shown CTI to reduce the number of days of homelessness, prevent new episodes of homelessness [10], diminish negative symptoms of schizophrenia [15], and reduce the risk of rehospitalisation [16]. Previous research also suggests that CTI is cost-effective [17]. To our knowledge, there is no research available on the effectiveness of CTI concerning abused women. However, CTI has proved to be effective for homeless mothers and children in reducing the time families were homeless and improving school and mental health outcomes among the children [18].

### Victims of intimate partner violence

Intimate Partner Violence (IPV) is a major public health problem in many countries throughout the world [19]. Victims often experience an accumulation of problems related to violence, poverty and social exclusion, which has numerous negative consequences for their health. Examples of frequently experienced adverse physical health consequences are severe injuries [20], chronic pain [21], gastro-intestinal problems [22] and gynaecological problems [23]. Furthermore, IPV can result in several psychological problems, such as depression [24], post-traumatic stress disorder (PTSD) [25] and substance abuse [26]. Frequently, IPV also leads to significant social isolation [27].

Estimates of the prevalence of women seeking refuge in shelters are rare. In one study it was estimated that 56,308 women and their children sought refuge in a shelter worldwide on any given day in 2011 [28]. These women report more severe abuse, related injury [25] and PTSD symptoms [29] than the victims who do not seek refuge in shelters. Although victims consider shelters as the most supportive and helpful means to deal with past experiences of violence [30] and their stay in shelters has positive effects on their well-being [30-32], research shows that women leaving shelters encounter many problems. These problems complicate their transition from the shelter to community living (with or without their (ex-)partner). Women indicate they have needs concerning obtaining resources [33,34], financial problems [3,35], information on and access to community services [3] and specialised treatment for depression and symptoms of PTSD [3,35]. Continuity of care is therefore essential and may help prevent re-victimisation and ensure improvements in the well-being of these women [3,36].

Several studies have demonstrated the lack of evidence concerning the effectiveness of treatments and interventions for victims of IPV [6,37]. In an extended international review Jonker, Sijbrandij, Van Luijtelaar, Cuijpers and Wolf (unpublished data, December, 2012) investigated the existing studies on the effectiveness of delivering shelter and post-shelter interventions to abused women. The results concerning post-shelter interventions, mainly consisting of advocacy services for women [34,38], are encouraging: interventions can diminish recurrence of violence, help end relationships (if wanted) and assist in successfully obtaining the resources required [39]. Furthermore, women experience a higher quality of life and are more satisfied with their social support due to post-shelter interventions [39,40]. Family post-shelter interventions consisting of instrumental and emotional support and teaching mothers child management skills were also evaluated and show promising results [41,42]. Nevertheless, as Jonker et al. (Jonker, Sijbrandij, Van Luijtelaar, Cuijpers, & Wolf, unpublished data, December, 2012) and Ramsay et al. [6] point out, the results of these studies must be interpreted with caution for a few reasons: the studies are few in number, some results are the outcome of only one study, and the quality of some of the studies is not clear. Also, there is an overall lack of standardised instruments in the studies. Evaluating the effectiveness of interventions for abused women after shelter exit therefore remains important, as underlined by several authors [6,37].

### Homeless people

Homelessness is often related to multiple and complex problems such as unemployment, financial problems and domestic conflicts [9,43,44]. In addition, physical complaints [45] and mental disorders [46] are more prevalent among homeless people than among the general population. Alcohol and drug dependence are the most common mental disorders, with 40% of the homeless population suffering from the former and 25% suffering from the latter. Furthermore, diagnoses of major depression (11%), personality disorders (23%), and psychotic illness (13%) have been frequently established among homeless persons [46].

Shelter services commonly require homeless people to transition through shelters and temporary housing situations before they become eligible for permanent independent housing in the community. These transitional housing facilities generally feature longer stays and offer a wide array of services for their residents (e.g. (mental) health, employment, legal and childcare services). After the transition to community housing, formerly homeless persons have to rely on conventional social welfare and health services again [47]. People who have made the transition to independent housing often experience readmission to shelter services, probably due to insufficient income, inappropriate housing, inadequate skills and the absence of ongoing support in the community [48,49]. To overcome the problems of long shelter stays and readmission to shelters, community-based programs have received more attention in recent years [7,50]. Research indicates that evidence-based interventions which provide temporary assistance with transitioning out of an institutional living environment could enable most homeless people to improve their living conditions and stabilise their lives [4,47].

Recently, De Vet et al. (De Vet, Van Luijtelaar, Brilleslijper-Kater, Vanderplasschen, Beijersbergen & Wolf, unpublished data, May, 2013) conducted a systematic review of the existing literature on the effectiveness of case management models which have been recommended for homeless clients and have been widely implemented. Results showed that both assertive community treatment (ACT) and CTI are cost-effective approaches. Because ACT is a model which advocates ongoing, comprehensive service provision by a multidisciplinary team which is accessible 24 hours a day, it is generally regarded as most appropriate for homeless persons with the greatest service needs (i.e. those with severe mental illness and/or substance abuse problems) [51]. CTI, on the other hand, seems to be applicable in a variety of settings serving homeless populations with less severe problems, due to its practical and time-limited nature [4]. In addition, this is the only model which was specifically designed for homeless persons who are at critical transitions in their lives [15]. CTI has proved to be effective in decreasing time spent in shelters and increasing time spent in stable housing as well as in reducing psychiatric symptoms and substance use for severely mentally ill homeless men and for homeless veterans with a serious mental illness (De Vet, Van Luijtelaar, Brilleslijper-Kater, Vanderplasschen, Beijersbergen & Wolf, unpublished data, May, 2013). Whether CTI can be effective for other subgroups of homeless people, in settings outside the United States and for outcomes such as quality of life, physical health and received social support, remains to be seen.

### Current studies

CTI is expected to be an appropriate intervention for abused women and homeless people making the transition to community living after staying in a shelter facility. Therefore, the Academic Collaborative Centre for Shelters and Public Mental Health and the Netherlands Centre for Social Care Research decided to modify the intervention for the Dutch context and to investigate the effectiveness of CTI for abused women and homeless people leaving shelter facilities in two randomised controlled trials (RCTs).

The primary research questions of these two studies are:

1. Is CTI more effective than care-as-usual with regard to improving quality of life for clients leaving women’s shelter facilities to move into supported or independent housing?

2. Is CTI more effective than care-as-usual with regard to preventing recurrent loss of housing for clients leaving homeless shelter facilities to move into supported or independent housing?

## Methods

### Study design

The studies are single-blinded, multi-centre RCTs. The studies comply with the criteria for studies which have to be approved by an accredited Medical Research Ethics Committee (aMREC). Upon consultation the Medical Review Ethics Committee region Arnhem-Nijmegen concluded that ethical approval was not necessary (registration numbers 2010/038 and 2010/247).

#### Experimental condition: critical time intervention

In the experimental condition participants receive CTI [10]. CTI is a time-limited, strengths-based psychosocial model for socially vulnerable people which serves to bridge the gap during the transition from institutional to community living. The intervention has two major goals. The first is to provide the client with emotional and practical support during this time of transition. The second is to maintain continuity of care by developing and strengthening links with people in the client’s personal and professional support system. At the end of the intervention period, the care provided by the CTI worker will have become redundant: the responsibility of care has gradually been passed to the client’s support system.

CTI starts with a preparatory phase (pre-CTI) in the shelter facility. During this phase the client and CTI worker become acquainted and the CTI worker begins to engage the client in a working relationship. The CTI worker also arranges a meeting with the client’s shelter case manager for a transfer of care. The preparatory phase ends when the client transitions to community living. The intervention is subsequently delivered in three phases of three months each: (1) Transition to the community, (2) Try-Out, and (3) Transfer of Care.

During the first phase, the CTI worker provides intensive emotional and practical support and assesses the client’s resources for the transition of care to the community. Performing a Risk and Needs Assessment helps to choose one to three focus areas which are considered most critical for the client’s community survival. These areas form the focus of attention during the intervention. Furthermore, in a Strengths Assessment [52], individual strengths and resources of the client are mapped and registered. The Strengths Assessment and the Risk and Needs Assessment form the starting point for the Personal Recovery Plan [52], in which the client’s goals and means to reach them are documented. During the intervention the Strengths Assessment and the Personal Recovery Plan will be continuously updated. During the first phase the CTI worker also accompanies the client to appointments with community providers to facilitate the development of lasting ties with them. The CTI worker also introduces the client to new care providers. At the end of the phase the CTI worker convenes a network conference with the community providers from the client’s personal and professional support system. One purpose of this meeting is to encourage all members of the client’s support system to communicate with each other.

During the second phase, the emphasis is on testing and adjusting the client’s support system. Around this time the CTI worker will defer (some of) his or her responsibilities to members of the client’s support system, as they should be capable of taking primary responsibility for the provision of support and services to the client by this point. The CTI worker maintains regular contact and encourages the client and his or her support system to handle problems independently. Modification of the support system by the CTI worker often proves to be necessary during this phase.

The final phase primarily focuses on completing the transfer of responsibility to community providers. The CTI worker has to ensure that the members of the support system meet together and agree on their roles in the ongoing support system. At the end of the phase the CTI worker takes leave of the client and terminates the contact. For a more detailed description of the intervention, see Herman and Mandiberg [4].

In the present studies, each of the participating organisations was required to assign at least two case managers for a three-day CTI training provided by a certified trainer. The case managers had to meet specific requirements: they needed to have a higher vocational education and broad experience in executive work with socially vulnerable people, preferably in women’s shelters and homeless shelters. Organisations were also required to assign an internal coach who was responsible for monitoring the model integrity of the intervention. The CTI workers and the internal coach met fortnightly to discuss the CTI clients and possible problems concerning the working conditions of the CTI workers, such as their caseload. The internal coach monitored the execution of the intervention with the aid of a CTI monitor record. The CTI workers documented their activities in a CTI record for each client. With the aid of these CTI records and the CTI monitor records, we are able to measure the CTI model integrity (Conover & Herman, unpublished Fidelity Manual, June, 2012). Consequently, we are able to link the results of CTI to the extent to which the intervention was carried out in accordance with the model. During the data collection period, centralised training sessions and work meetings were frequently organised by the researchers to support CTI workers in carrying out the intervention according to the model.

#### Control condition: care-as-usual

Participants in the control condition received care-as-usual. This could include care from the shelter organisation or a referral to community services like social services and mental health services. The type and duration of the provided care by organisations varied widely. During the data collection period, we collected information about the type of services participants in the control condition received. To reduce the risk of contamination between conditions, participants in the control condition did not receive any services from the CTI workers. Participants were allowed to withdraw from the study at any time without jeopardising their support.

### Selection of recruitment settings and clients

Shelter facilities were eligible for selection as recruitment settings if their organisation was participating in the Academic Collaborative Centre for Shelters and Public Mental Health in 2009. Furthermore, shelter facilities needed to provide 24-hour services, serve at least 50 different clients aged 18 or older per year, and expect to continue their services over the next five years. They also had to provide services to clients who usually spend no longer than 12 months in the shelter facility. An additional criterion for shelter facilities for abused women was that they needed to provide care to clients who usually spend at least six weeks in the shelter. Shelter facilities were not included in the selection if their services were limited to a specific group of clients, such as drug users or teenage mothers, or if they already offered care which was very similar to CTI.

Out of the shelters matching these criteria and willing to participate, we selected at least one and at most four facilities per organisation and ensured selected shelter facilities were distributed evenly among the four regions of the Netherlands (north, east, south and west). Nineteen women’s shelters (run by nine organisations) and 22 shelters for homeless people (run by nine organisations) participated in the studies. These shelter facilities for homeless people could be best characterised as transitional housing facilities. Some participants recruited in these facilities have not experienced literal homelessness, seeing that not all of them have spent time on the street, in emergency shelters or doubled-up with family or friends before entering these facilities. However, the participants may be characterised as homeless persons using a broader definition; all participants entered these transitional housing facilities because they lacked the resources or support networks needed to obtain housing [53].

Clients were eligible for participation if they:

• were aged 18 years or older;

• were willing to receive support from the shelter organisation during and after leaving the shelter facility;

• were staying in the shelter facility due to intimate partner or honour-related violence (abused women);

• had been staying in the shelter facility for at least six weeks (abused women);

• would be staying in the shelter facility for no longer than 14 months at the date of departure (homeless people);

• had been given a date of departure from the shelter facility or had received a declaration of urgency;

• were going to depart to supported or independent housing where no daily supervision or support would be present; and

• would be required to pay rent or housing costs in their new accommodation.

Clients were unable to participate if they were going to live in a region where none of the participating organisations provided services. Intellectual disabilities, psychological problems, drug use or lack of command of the Dutch language did not constitute exclusion criteria.

### Data collection period and recruitment procedure

Inclusion of participants in both studies began in December 2010. Clients in women’s shelter facilities were included until July 2012 and clients in homeless shelter facilities until December 2012. Clients were informed about the studies through leaflets and information letters they received from the staff. In some shelter facilities clients were also informed during regular client meetings, which were occasionally visited by a research assistant.

Each shelter facility appointed a contact person who registered clients who were about to leave the shelter and screened them for eligibility. Clients eligible to participate were asked to provide permission for their contact details to be passed on to the researchers. If clients gave permission, a research assistant contacted them by telephone to provide additional information about the study and to ask them to participate. After clients had indicated they were willing to participate and the research assistant had checked again whether clients met the selection criteria, the research assistant scheduled an appointment for the first interview. This interview took place in the shelter facility or, if this was not possible, it was conducted in a public place, such as a library. Before the first interview was executed, clients had to provide written informed consent.

### Randomisation and blinding

Randomisation was stratified according to shelter location. Before the start of these studies two allocation schedules were generated using the Random Integer Set Generator at http://www.random.org/integer-sets, which employs a restricted randomisation procedure. The random numbers were secured in a digital file and concealed until conditions were assigned. To avoid periodic imbalance, block randomisation was used: out of every four participants, two were randomly assigned to the experimental group. One of two researchers allocated the participants after the first interview and depending on the condition to which a client was allocated, a CTI worker or case manager initiated the assigned care.

Shelter staff, including the contact person who screened clients for eligibility, did not have any foreknowledge of condition assignment; they were informed about the assigned condition after the first interview had been conducted. Clients agreed to participate in the study before randomisation and without knowing in which condition they would be enrolled. They were blinded to allocation until they met their CTI worker or case manager after the first interview. The researchers withheld information about allocation from the research assistants, who conducted the interviews with participants, to ensure that these assistants were blinded from condition assignment.

### Baseline and follow-up measurements and retention rates

**Figure 1:**
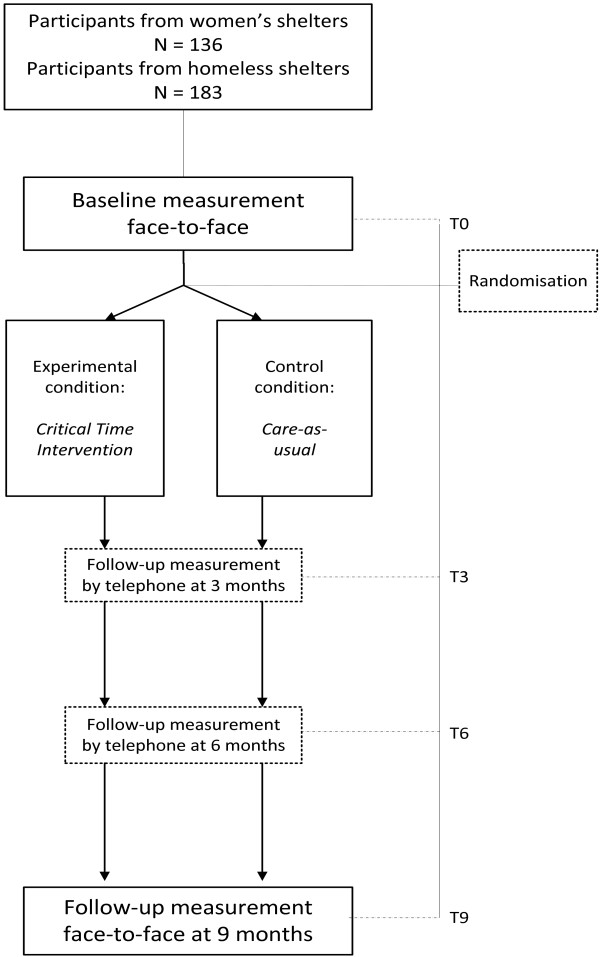
Flowchart of the RCTs and measurements.

Outcome measurements took place before participants left the shelter facility and at three, six and nine months after shelter exit (see Figure 1). Ideally, the first interview was planned up to four weeks before leaving the shelter facility, because the preparatory phase of CTI should be initiated before the transition. If this was not possible, the interview took place up to two weeks after shelter exit. The second and third interviews were held by phone.

In order to maximise retention rates, several arrangements were made. Research assistants were selected using stringent criteria: they had to be able to approach participants positively and create positive rapport with them. Furthermore, research assistants collected contact information of people in participants’ networks to track participants for follow-up. Participants received interview payments increasing over time from €15 at baseline to €30 at nine-month follow-up. After each interview they also received an appointment card [54] with the date of the next interview and a post card to thank them for participation. Finally, if participants were difficult to track, interviews were sometimes still conducted even long after the follow-up moment had passed, because having a late interview was considered preferable to having no interview at all [54].

### Research assistants

The measurements consisted of structured interviews administered by trained research assistants. They all had relevant academic or vocational degrees and preferably experience in working with socially vulnerable people. Several multilingual research assistants were recruited for the most common languages and, if necessary, the interviews were conducted with the aid of an interpreter. Interviews with female participants were conducted by female research assistants only. The CTI workers and case managers were not involved in the interviewing process, although they sometimes assisted by scheduling appointments with participants.

### Primary outcome measures

The primary outcome of the study with clients transitioning from shelter facilities to community living is recurrent loss of housing, as measured by the number of days participants do not live in conventional independent housing (property or legal (sub)tenancy) or accommodation permanently provided by friends, relatives or acquaintances without legal (sub)tenancy during follow up.

In addition, the effect of CTI compared to care-as-usual will be determined based on the number of days participants are:

• literally homeless:

▪ living rough, i.e. living on the streets or in public spaces, without a shelter which can be defined as living quarters;

▪ staying in an emergency shelter or night shelter; or staying with friends, relatives or acquaintances temporarily.

• institutionalised:

▪ residing in transitional accommodation (where the period of stay is intended to be short-term), residential care or supported accommodation (long stay) for homeless people or women’s shelter accommodation;

▪ living in residential care or supported accommodation for people with mental health or substance abuse problems;

▪ staying in a medical institution, drug rehabilitation institution or psychiatric hospital; or

▪ staying in a correctional or penal institution.

• marginally housed:

▪ residing in temporary accommodation, not intended as place of usual residence (hotels, motels, inns, boarding houses, pensions, rooming houses or other lodging houses); or

▪ staying in non-conventional accommodation, not intended as place of usual residence (squatted buildings, mobile homes, summer houses or buildings due for demolition).

To assess participants’ residential histories, the six-month Residential Follow-Back Calendar [55] was included in the questionnaire. For the purpose of the study, the duration of the calendar was adapted to three months. Research assistants first determined participants’ current living situation and then worked backward day by day to the date of the preceding interview. Based on events significant to participants (such as birthdays and holidays), research assistants registered all living situations and calculated the time spent in each location. The Residential Follow-Back Calendar has a high test-retest-reliability, with coefficients, except for one residential outcome measure, consistently ranging from .80 to .93. The concurrent validity, investigated with agency data and self-reports, is also high, with coefficients ranging from .84 to .92 [56].

The primary outcome of the study in women’s shelters is quality of life. This outcome was assessed using the brief version of Lehman’s Quality of Life Interview (QoLI) [57-59]. Both objective (what participants do and experience) and subjective (their feelings about the experience) quality of life were determined in eight life domains: living situation, daily activities & functioning, family, social relations, finances, work & school, legal & safety issues, and health. Objective quality of life describes the actual living situation of the participants, such as the type of accommodation, current employment, sources of income and arrest history. Subjective quality of life was scored on a seven-point Likert scale, ranging from *terrible* to *delighted*. The QoLI also contains a global measure of life satisfaction, which was administrated at the beginning and at the end of the interview. The psychometric properties of the brief version of this instrument are good and comparable to the full interview version [60].

Quality of life is a secondary outcome in the homeless shelter study and was also examined using the brief version of Lehman’s Quality of Life Interview (QoLI) [57-59]. The QoLI is commonly used to assess quality of life in studies with homeless participants [61,62].

### Demographic variables

Demographic variables included age, gender, nationality, country of birth (of participants and their parents), residence permit status, religion, marital status, household composition and educational level. Participants leaving women’s shelter facilities who were married or divorced were asked if their marriage had been arranged (for example, by family members).

### Secondary outcome measures

The following secondary outcomes were measured in both studies:

• Care needs were assessed using an instrument developed for these studies. For seven domains, participants were asked to indicate whether they wanted help, whether they received help in this area, and whether this was the right help. The domains, which have been subdivided into several items, are housing & daily life (finding housing, maintaining housing, household care, self-care), finances & daily activities (finances, daily activities and recreation, finding work, literacy, transport/mobility), physical health (physical health, alcohol abuse, drug abuse, dental care, nourishment), mental health (mental health, empowerment), safety & protection against violence (protection of own safety, protection of others against own behaviour), social relations (relationship with family, social contacts with friends or acquaintances, relationship with partner), and children (help with contact with children, help for children). Response categories were adopted from the short form Quality of Life and Care (QoLC) [63].

• Self-esteem was evaluated using the Dutch version of the 10-item Rosenberg Self-Esteem scale (RSES) [64,65]. Answers could be given on a four-point scale ranging from *strongly agree* to *strongly disagree*. The RSES shows satisfactory reliability and validity with samples of abused women, with alphas generally between .80 and .90 [66,67]. This instrument has also been used in several studies among homeless people and shows high internal consistency [68].

• The De Jong Gierveld & Kamphuis Loneliness Scale [69] was used to measure loneliness. The scale consists of five positive and six negative items, which are answered with *yes*, *more or less* or *no*. One of the positive items is ‘There are many people I can trust completely’ and a negative item is ‘I miss having people around me’. This scale has been applied in several studies among a range of populations and shows sufficient reliability and validity [70].

• Social support was measured by five items used in the RAND Course of Homelessness Study [71], which were derived from scales developed for the Medical Outcome Study (MOS) Social Support Survey [72]. Participants were asked to indicate how often different kinds of support were available to them through family, friends or other acquaintances, and a partner (if applicable). For each potential source of support, they were asked how often this source was available to (1) have a good time with, (2) provide them with food or a place to stay, (3) listen to them when they were talking about themselves or their problems, (4) accompany them to an appointment to provide moral support, and (5) show that he or she loves and cares for them. A five-point scale ranging from *none of the time* to *all of the time* was used to score the perceived support. The MOS Social Support Survey has been used in several studies among homeless people [73,74]. The 19-item survey showed high convergent and discriminant validity and internal consistency reliability coefficients range from .91 to .97 for the four subscales [72].

• The frequency and intensity of substance use was measured with items from the Dutch translation of the European version of the Addiction Severity Index (EuropASI) [75,76]. The Addiction Severity Index has frequently been used in surveys among homeless people with serious mental and/or addiction problems [77,78]. Studies among substance-abusing populations show satisfactory results for the reliability and validity of the EuropASI [79].

• The Dutch version of the Brief Symptom Inventory (BSI-53) [80,81] was used to assess psychological distress. The BSI consists of 53 items covering nine symptom dimensions: somatisation, obsession-compulsion, interpersonal sensitivity, depression, anxiety, hostility, phobic anxiety, paranoid ideation and psychoticism. Items are measured on a five-point Likert scale, ranging from *not at all* to *extremely*. The BSI has widely been used in research among homeless people and abused women to measure mental health [82,83]. The Dutch version of the BSI demonstrates good reliability and validity [81].

• Service use was evaluated with a self-constructed questionnaire used in several studies by the Netherlands Centre for Social Care Research. Participants were asked to indicate whether they had used the services of certain care providers (e.g. general practitioner, dentist and social services) in the past nine months and in the past 30 days.

• To assess the working alliance between participants and professionals the short version of the 36-item Working Alliance Inventory (WAI) [84-86] (Flemish translation) was used. We simplified the description of items to make them comprehensible for our target groups. The WAI is commonly used in psychotherapy to assess therapeutic alliance and has been extensively studied in this context [87]. Participants assessed the working alliance with their CTI worker or case manager on a seven-point Likert scale ranging from *never* to *always*. The validity and reliability of the English version of the 36-item WAI has been demonstrated to be good [88].

• Experiences with shelter and community care services are evaluated with the Consumer Quality Index for Shelter and Community Care Services (CQI-SCCS) [89]. The CQI-SCCS consists of four scales: ‘Living Conditions’ (six items), ‘Client-Worker Relationship’ (four items), ‘Services Received’ (10 items), and ‘Results of Services’ (five items). The items are scored on a four-point scale ranging from *never* to *always*. Construct validity of the CQI-SCCS is good and test-retest reliability sufficient [89].

In the women’s shelter study the following secondary outcomes are also measured:

• Re-abuse was measured with one single question: ‘Have you been abused since the last interview?’ Answers were *yes* or *no*. Participants were also asked what kind of violence they experienced: 1) ‘physical violence, like being beaten, or he or she threw objects at you’, 2) ‘sexual violence, like intimacy you didn’t want or like’ and 3) ‘emotional violence like threats, or he or she had no respect for your feelings, or said things to hurt you’. Answers were *yes*, *no* or *no information*.

• Symptoms of depression in the week prior to the interview were measured using the Dutch version of the Centre for Epidemiological Studies Depression scale (CES-D) [90,91], a well-established measure of depressive symptomatology. This instrument has been used in several studies among abused women [92,93]. The CES-D contains 20 items rated on a four-point scale ranging from *seldom or never* to *always or almost always*. The CES-D demonstrates good internal reliability, with coefficients ranging from .87 to .93 in research among a general population [90]. Additionally, CES-D demonstrates convergent validity, because it correlates with other measures of depression, as well as sound construct validity [90]. The Dutch version of the CES-D demonstrates acceptable psychometric qualities [91].

• Post-traumatic stress symptoms were measured using the Dutch version of the Impact of Event Scale (IES) [94,95]. The IES is a self-report instrument used in studies among abused women [25,96] and measures changes in reaction to potentially traumatic events. It consists of 15 items, seven of which are related to intrusion (e.g. thinking about the event despite not wanting to do so or having dreams about it) and eight are related to avoidance (e.g. staying away from reminders of it or not wanting to talk about it). The items of the Dutch version of the IES are scored on a four-point scale: *not at all*, *seldom*, *sometimes* and *often*. The Dutch IES has adequate internal consistency with reliability coefficients varying from .87 to .96 for the total score [97].

• Parenting Stress was assessed using two scales of the experimental version of the Parenting Stress Index [98]: ‘Problems with parent–child relationship’ and ‘Problems with parenting’. These two scales contain 22 items rated on a four-point scale ranging from *not true* to *very true*. Two of the items are ‘When I am with my child I feel good’ and ‘I know I am doing a good job as a parent’. The instrument demonstrates good construct validity and good reliability [98].

### Sample sizes

The required sample size for the women’s shelter study is based on the amount of improvement in the subjective quality of life score resulting from the intervention. No information is available from earlier experimental research about what amount of improvement is to be expected. In a study of advocacy services for women leaving shelters, a small but statistically significant difference was found on a similar subjective quality of life scale. At the 12-month follow-up, an effect size of .2 was found: women in the experimental group reported a mean score of 5.08 (SD=1.01) while controls reported 4.87 (SD=1.04) [39]. Since the duration and intensity of the intervention tested in the current trial are much greater than those of the advocacy intervention, a much larger improvement in the subjective quality of life score is to be expected. To calculate the required sample size, we assumed a clinically relevant effect size of .55, resulting in a mean improvement in the subjective quality of life score of .8 (SD=1.5). To detect this difference with 80% power (α = .05, two-sided), each group should contain 55 participants. We considered a potential loss of power due to clustering of data in case managers. Assuming an intra-class correlation coefficient (ICC) of .05 and an average number of three clients per case manager, the number of participants in the experimental and control group should both be increased to 61. Accounting for 30% attrition, the intended sample size is 174 for the total group.

Regarding the homeless shelter study, the sample size calculation is based on the difference between groups in the proportion of participants who become homeless. The expected mean difference is based on a study conducted by Susser et al. [10], who compared clients with severe mental illness receiving CTI in addition to care-as-usual when transitioning to community living to controls receiving care-as-usual only. In the last month of the 18-month follow-up 8% of the group receiving CTI was homeless compared to 23% of the controls. Each group should contain 91 participants to detect this difference with 80% power (α = .05, two-sided) in the current study. Assuming the ICC to be .05 due to clustering of clients in case managers and an average number of five clients per case manager, the number of participants in each group should be increased to 109. Accounting for 30% attrition, the intended sample size is 312 for the total group.

### Analyses

Statistical analyses will be performed using the software program IBM SPSS Statistics for Windows, Version 20.0. Differences in baseline characteristics (e.g. age, gender, education, nationality) between the intervention and control groups will be checked. The primary outcomes of the studies (improvement of quality of life during the nine-month follow-up and decrease in days unhoused over nine months), as well as other outcomes, will be compared between groups according to the intention-to-treat principle using mixed-effects analyses to correct for clustering. Quality of life at baseline will be added as covariate. Dichotomous outcomes will be compared using multi-level logistic regression and odds ratios will be reported. In the analyses corrections will be made for important characteristics such as age, gender, duration of victimisation, duration of homelessness and severity of psychological problems.

## Discussion

Although it is widely acknowledged that socially vulnerable people such as abused women and homeless people leaving shelter facilities merit appropriate care in the community [34,35,47], there is a notable lack of evidence-based interventions which systematically address the needs of these populations. As a result, abused women often experience re-victimisation [39] and formerly homeless people often experience readmission to shelter services [49].

Research in the United States has shown CTI to be effective for different populations. To examine whether CTI is also an appropriate intervention for socially vulnerable people in The Netherlands, the intervention was modified for the Dutch situation. The present studies are unique because the effectiveness of interventions for these two groups has not been investigated in RCTs before in The Netherlands, and rarely outside the United States [99] (De Vet, Van Luijtelaar, Brilleslijper-Kater, Vanderplasschen, Beijersbergen & Wolf, unpublished data, May, 2013; Jonker, Sijbrandij, Van Luijtelaar, Cuijpers & Wolf, unpublished data, December, 2012).

The studies have several strengths. Outcomes are mainly measured with standardised instruments commonly used in research among these populations and provide relevant information on the participants. Due to their specific socio-demographic characteristics (e.g. poverty, homelessness, legal status), the participants of the target populations are known to be difficult to follow up [100]. Therefore, we administered several proven effective measures to minimise attrition: frequent contacts with participants, short follow-up periods, careful selection and extensive training of research assistants, precise record keeping and distribution of appointment cards [54,100,101]. Consequently, follow-up rates in these studies are expected to be satisfactory. An additional strength of the studies is that the participants included will probably form an accurate reflection of the Dutch shelter population due to the participation of shelter facilities from different regions, the inclusion of clients with alcohol or drugs dependence and the inclusion of clients with a poor understanding of the Dutch language. In other studies these factors have often been reasons for non-participation [102,103]. A last strength is that we measure the model integrity of CTI: the effects of CTI found in these studies, or the absence of effects, can be linked to the extent to which the intervention was carried out in accordance with the model.

Some characteristics of these studies should be considered when interpreting the results. Although there is a wide variety of clients in women’s shelter facilities, only victims of intimate partner or honour-related violence were selected for the study in women’s shelters in order to establish a homogeneous group of participants. For this reason, statements about other specific groups in women’s shelter facilities, such as victims of human trafficking, cannot be made. Additionally, it should be noted that the effects of CTI are expected to be most pronounced over the longer term (after the first nine months, i.e. the treatment period) [10]. The interviews in our studies are conducted at several time points, with the last measurement nine months after shelter exit. Therefore, it is not possible to determine possible effects for the long run. More extensive longitudinal studies are needed to examine these effects.

Due to the lack of evidence on effective interventions for abused women and homeless people, the results will extend the evidence for the effectiveness of CTI applied to different populations in countries outside the United States. Concurrently, if CTI proves to be effective, the studies will offer shelter organisations in The Netherlands the opportunity to include this case management model in their professional standards and improve the (quality of) services for clients.

## Competing interests

The authors declare that they have no competing interests.

## Authors’ contributions

JW and AvH are responsible for the design of the studies. MB supervises the execution of the studies. DL and RdV are responsible for collecting data, performing statistical analysis and reporting of study results. They wrote the manuscript supervised by MB, AvH, DH and JW who commented critically on the manuscript. All authors read and approved the final manuscript.

## Pre-publication history

The pre-publication history for this paper can be accessed here:

http://www.biomedcentral.com/1471-2458/13/555/prepub
